# *IFNGR1 *single nucleotide polymorphisms in rheumatoid arthritis

**DOI:** 10.1186/ar1927

**Published:** 2006-03-23

**Authors:** Stefan Mattyasovszky, Alla Skapenko, Joachim R Kalden, Peter E Lipsky, Hendrik Schulze-Koops

**Affiliations:** 1 Nikolaus Fiebiger Center for Molecular Medicine, Clinical Research Group III, University of Erlangen, Germany; 2 Department of Internal Medicine III, University of Erlangen, Germany; 3 National Institute of Arthritis and Musculoskeletal and Skin Diseases, Bethesda, Maryland, USA; 4 Department of Trauma Surgery, University of Mainz, Germany

## Abstract

On the basis of their biological function, potential genetic candidates for susceptibility to rheumatoid arthritis can be postulated. *IFNGR1*, encoding the ligand-binding chain of the receptor for interferon gamma, IFNγR1, is one such gene because interferon gamma is involved in the pathogenesis of the disease. In the coding sequence of *IFNGR1*, two nucleotide positions have been described to be polymorphic in the Japanese population. We therefore investigated the association of those two *IFNGR1 *single nucleotide polymorphisms with rheumatoid arthritis in a case-control study in a central European population. Surprisingly, however, neither position was polymorphic in the 364 individuals examined, indicating that *IFNGR1 *does not contribute to susceptibility to rheumatoid arthritis, at least in Caucasians.

## Introduction

Many pathologic autoimmune responses are characterized by an imbalance in the T helper type (Th) 1/Th2 ratio in favor of the former [1]. As activated Th1 cells mediate their functions via their signature cytokine, interferon gamma (IFNγ), the interferon gamma receptor (IFNγR) plays an important role in the pathogenesis of these diseases by transmitting IFNγ signaling. The IFNγR consists of the ligand-binding chain IFNγR1 and the signal-transducing chain IFNγR2. Within the coding region of the *IFGR1 *gene [GeneBank accession number NM_000416], two single nucleotide polymorphisms (SNPs) (40 C/T and 1,400 T/C) that result in the amino acid substitutions valine to methionine at position 14 (V467M) and leucine to proline at position 467 (L467P), respectively, have been identified in the Japanese population [2,3].

Th1 cells have been implicated in many aspects of the pathogenesis of rheumatoid arthritis (RA) [1]. Evidence suggests that both genetic and environmental factors contribute to the development of rheumatoid inflammation [4-6]. Elucidating the genetic basis of RA, however, is still one of the major challenges in modern rheumatology. The identification of RA susceptibility genes has been difficult because RA is a complex autoimmune disease that, unlike classic Mendelian traits causally related to highly penetrant rare mutations of single genes, appears to be caused by small individual effects of many poorly penetrant common alleles.

The association of the two *IFNGR1 *SNPs 40 C/T and 1,400 T/C with susceptibility to immune disorders mediated by an imbalance in the Th1/Th2 ratio has recently been demonstrated in Japanese cohorts; for example, in allergy [2] and in systemic lupus erythematosis [3,7]. Because of the potential importance of *IFNGR1 *SNPs in immunity in health and disease in people of all ethnic origins, these observations prompted us to perform a case-control association study to investigate the role of both *IFNGR1 *SNPs in susceptibility to RA, a Th1-mediated autoimmune disease, in a Caucasian population.

## Materials and methods

One hundred and one patients with an established diagnosis of RA, according to the 1987 revised criteria of the American College of Rheumatology for the classification of the disease, were enrolled in the study. The 101 patients represented an ethnically homogeneous cohort of Caucasian RA patients. The median (range) age of the patients at time of the analysis was 63 years (17–81 years), and 76% were female. A cohort of 171 healthy individuals matched on the basis of age, sex and origin were used as a healthy control group. All protocols and recruitment sites have been approved by the local institutional review boards, and all subjects were enrolled with informed written consent.

Genomic DNA was isolated from white blood cells using the AquaPure Genomic DNA isolation kit (BioRad Laboratories, Munich, Germany). Genotype analysis was performed by allele-specific PCR and verified by direct sequencing. PCR primer and probe sequences for the V14M SNP were 5' -AGTGGAGTGGCTACAAAGGTCCC-3' (forward primer) and 5' -CCCATCTCAGCCCTGCTCAC/T-3' (reverse primer). PCR primer and probe sequences for the L467P SNP were 5' -CATGTGCTAGTGGATCTACT/C-3' (forward primer) and 5' -AGTGGAGTGGCTACAAAGGTCCC-3' (reverse primer).

## Results and discussion

In marked contrast to previous findings, no polymorphic alleles (neither thymine at position 40 nor cytosine at position 1,400) were detected in any of the individuals tested. This was surprising because both positions were highly polymorphic in the original publications. In those publications, heterozygosity at position 40 (V14M) was detected in four individuals (4.4%) in a small cohort of 91 healthy controls and even in 15 out of 96 (15.6%) lupus patients [7], and heterozygosity at position 1,400 (L467P) was detected in four individuals (6.7%) in a cohort of 89 allergic patients, although it was absent in healthy controls [2].

To verify our results for position 1,400, therefore, we additionally analyzed genomic DNA of 82 well-characterized atopic patients with an established clinically relevant type I allergy directed, for example, to house dust, mite, birch pollen or bee venom. However, this population was not polymorphic at either of the two positions either. Our data therefore strongly suggest that the *IFNG1R *gene is not polymorphic at those two positions at least among Caucasians and therefore does not contribute to genetic susceptibility to RA.

Some ethnic variations in the frequencies of SNPs linked to RA have been already reported [8]. Analysis of RA-associated SNPs in solute carrier family 22 members 4 and 5 (*SLC22A4 *and *SLC22A5*) [9] and in protein tyrosine phosphatase (*PTPN22*) [10] in different ethnic groups revealed that the disease-associated polymorphic alleles usually common in Caucasians (over 8% prevalence) are absent or only extremely rarely present in the Japanese population [8]. Our data are in line with these observations and together implicate that association findings should be carefully analyzed in different ethnic contexts to allow meaningful conclusions regarding whether the gene of interest is of importance in the susceptibility to a particular autoimmune disease.

## Conclusion

*IFNGR1 *is not polymorphic in Caucasians although it is polymorphic in the Japanese population. It is therefore unlikely to contribute to susceptibility to RA, at least in Caucasian cohorts of patients.

## Abbreviations

IFNγ = interferon gamma; IFNγR = interferon gamma receptor; PCR = polymerase chain reaction; RA = rheumatoid arthritis; SNP = single nucleotide polymorphism; Th = T helper type.

## Competing interests

The authors declare that they have no competing interests.

## Authors' contributions

SM performed the experiments. AS participated in the design of the study and wrote the manuscript. JRK and PEL participated in the design of the study. HS-K participated in the design of the study and helped to draft the manuscript.
